# Pine Needle Extract Applicable to Topical Treatment for the Prevention of Human Papillomavirus Infection

**DOI:** 10.4014/jmb.2010.10055

**Published:** 2020-11-10

**Authors:** Hee-Jung Lee, Mina Park, HeeJae Choi, Aleksandra Nowakowska, Chiung Moon, Jong Hwan Kwak, Young Bong Kim

**Affiliations:** 1 Department of Biomedical Science and Engineering, Konkuk University, Seoul 05029, Republic of Korea; 2 Moonstech, Ansan 15588, Republic of Korea; 3 School of Pharmacy, Sungkyunkwan University, Suwon 16419, Republic of Korea

**Keywords:** HPV, pine needles, extract, antiviral, topical treatment

## Abstract

Most cervical cancers are associated with high-risk human papillomavirus (HPV) infection. Currently, cervical cancer treatment entails surgical removal of the lesion, but treatment of infection and preventing tissue damage are issues that still remain to be addressed. Herbal medicine and biological studies have focused on developing antiviral drugs from natural sources. In this study, we analyzed the potential antiviral effects of *Pinus densiflora* Sieb. et Zucc. leaf extracts against HPV. The pine needle extracts from each organic solvent were analyzed for antiviral activity. The methylene chloride fraction (PN-MC) showed the highest activity against HPV pseudovirus (PV). The PN-MC extract was more effective before, rather than after treatment, and therefore represents a prophylactic intervention. Mice were pre-treated with PN-MC via genital application or oral administration, followed by a genital or subcutaneous challenge with HPV PV, respectively. The HPV challenge results showed that mice treated via genital application exhibited complete protection against HPV. In conclusion, PN-MC represents a potential topical virucide for HPV infection.

## Introduction

Human papillomavirus (HPV) is a group of viruses that are extremely common worldwide. There are more than 100 types of HPV, including at least 14 cancer-causing viruses. Although the majority of infections cause no symptoms and are self-limiting, genital HPVs have become a major public health concern because persistent infection with certain types can cause cervical cancer, which kills about 311,000 women worldwide each year [1-3]. HPV serotypes 16 and 18 are reported to account for approximately 70% of cases, with the most common serotypes detected in women with cervical cancer [4].

Although commercial vaccines, including Gardasil and Cevarix, represent a remarkable improvement in the fight against cervical disease and other anogenital cancers, vaccinated individuals may remain infected with HPVs that are not targeted by the vaccines. Further, because the vaccines are relatively expensive, they may not be available to all women, especially those in developing countries [5, 6]. Currently, no FDA-approved antiviral drugs are available to treat HPV infection [7, 8].

Recent studies investigating the prevention and treatment of HPV related to cervical cancer and other genital diseases are attracting increasing attention worldwide [8]. The study of inhibitors is focused on the oncogenic proteins E6 and E7, and the replication proteins E1 and E2 [9, 10]. The therapies for HPV-associated lesions, including cryotherapy, surgical excision, and topical application of cytotoxic agents, are mostly ablative and cytodestructive although genital warts can be treated by topical application of the immunomodulator imiquimod [11, 12]. Despite studies investigating the treatment of HPV-related lesions, effective drugs or treatments for HPV infection are imperative.

Herbal medicine is considered one of the alternative approaches to the treatment of viral diseases (Ghanbari *et al*., 2019). Approximately 70,000 plant species are used in herbal medicine, including the treatment of HPV infection [13, 14]. Green tea extract has been reported to regulate the genes involved in the pro-inflammatory response to HPV infection and inhibit the binding to receptors [15]. A recently approved Polyphenon^®^E ointment found effective against HPV-related diseases was formulated with catechins extracted from green tea [16].

*Pinus densiflora* Sieb. et Zucc. has been reported to contain several phenolic compounds that exhibit biological activity, such as antibacterial and antioxidant effects, as well as potential antiviral activity against the influenza A virus [17]. Among studies on the pharmacological action of pine needles, saponin is known to exhibit strong lethal activity against cancer cell lines [18], but the inhibition of *P. densiflora* against HPV infection has yet to be reported. In the current study, we investigated the inhibitory activity of *P. densiflora* leaf extract against HPV in vitro and in vivo.

## Materials and Methods

### Cells

293TT cells were derived from 293T cells by incorporation of cDNA encoding for SV40 spliced T antigen (provided by John T. Schiller). The cells were cultured within Dulbecco’s modified minimal essential medium (DMEM) supplemented with 10% FBS and hygromycin B (400 μg/ml (Invitrogen, USA) at 37°C with 5% CO_2_ [19].

### Preparation of HPV16 Pseudoviruses (PVs)

HPV16 PVs were prepared as described previously via co-transfection of 293TT cells with p16L1/L2 plasmids and pSEAP or pLucf plasmid [20-23]. Cells were incubated at 37°C for 48 h, followed by lysis, and purification of PVs on an OptiPrep gradient [24, 25].

### Mice

Six-week-old female BALBb/c mice (Orient-Bio, Korea) were randomly divided into four groups, each comprising five mice. The use of animals in these experiments was approved by the Institutional Animal Care and Use Committee of Konkuk University (Approval No. KU12078).

### Extracts of Pine Needles

Gangwon-do pine needles (*Pinus densiflora*) were purchased at Gyeongdong Market (Korea). Pine needles were extracted with 95% ethanol (10 vol/wt). The extraction was repeated three times, followed by filtration of the extract solution and evaporation (PN-T). The extracts were suspended in distilled water and sequentially sorted into equal amounts of hexane, methylene chloride, ethyl acetate, and n-butanol. Using a separatory funnel, the hexane (PN-H), methylene chloride (PN-MC), ethyl acetate (PN-E), n-butanol (PN-B), and aqueous (PN-W) fractions were obtained.

### Cytotoxicity Assay

To determine the cytotoxicity of the pine needle extracts, the cell viability was measured using the water-soluble tetrazolium salt method with an EZ-Cytox Kit (Daeil Lab Service, Korea) according to the manufacturer’s instructions. The 293TT cells were seeded on a 96-well plate at a density of 1 × 10^4^ cells/well. The cells were cultured for 1 day, followed by treatment with serial dilutions of extracts and incubated at 37°C for 2 days. EZ-Cytox solution was added to each well and incubated for 2 h, followed by spectrophotometric measurement of absorbance at 540 nm.

### Screening of Antiviral Activity

The 293TT cells were grown in 96-well plates at 5 × 10^3^ cells/well for 24 h. Different extract concentrations were added to the cells for 16 h. The cells were gently washed with PBS once to remove the remaining extract inside the wells. The cells were infected with 1 × 10^5^ relative light units (RLU) of HPV16-SEAP PVs for 1 h. The medium was removed and replaced by a complete culture medium. The cultures were incubated for 48 h at 37°C under 5% CO_2_, and then the SEAP content in 10 μl of clarified cell supernatant was determined using the Great EscAPe SEAP Chemiluminescence Kit (Clontech, USA). Each concentration of the extract was assayed for virus inhibition in triplicate.

### Pre-Treatment Assay

Before virus inoculation, non-cytotoxic concentrations (100 μg/mlL and 50 μg/ml) of the extract were added to the cells and incubated for 4, 8, 12, or 16 h. The cells were gently washed with PBS once to remove the remaining extract inside the wells. HPV16-SEAP PV at a multiplicity of infection (MOI) of 20 was introduced for 1 h, and the cells were washed with PBS. The medium was removed and replaced by a complete culture medium. The cultures were incubated for 48 h at 37°C under 5% CO_2_. The supernatant following each pre-treatment was collected at 48 h after infection.

### Adsorption Assay

Cells were pre-adsorbed with HPV16-SEAP PV using different concentrations (100, 50, 25, 12.5, 6.25, or 3.125 μg/ml) of PN-MC extract for 1 h at 37°C. Cells were washed twice with PBS and replaced with culture medium. After 48 h, the antiviral effect by adsorption between virus and extract was evaluated using a SEAP Chemiluminescence Kit.

### Post-Treatment Assay

The cells were initially infected with HPV16-SEAP at a MOI of 20. The cells were washed with 200 μl of PBS after 1 h, followed by the addition of different concentrations (800 to 12.5 μg/ml) of extract to the cells, and then incubated for 48 h. After 48 h, the antiviral activity was determined via measurement of SEAP chemiluminescence.

### Challenge Test with HPV PVs

The mice were challenged with HPV PV, as described previously [26]. Seven days before in vivo challenge with PV, mice were synchronized in a diestrus-like status via subcutaneous injection with 3 mg of Depo-Provera (Pharmacia, Belgium).

The mice received the PN-MC treatment via genital application or oral administration. The genital administration entailed use of the extracts in the vaginal tract and treatment at 3 mg/mouse in 0.5% methylcellulose once a day for 3 days. The oral dose of the extract was 6 mg/mouse in 0.5% methylcellulose (Sigma-Aldrich, USA) once a day for 5 days. Four hours after the last administration of the PN-MC extract, a challenge test of each mouse was performed.

The genital treatment involved challenge with 1 × 10^7^ infectious forming units (IFU) of HPV16-Lucf PV, each in a 20 μl solution containing 4% carboxymethylcellulose (treatment involved a subcutaneous (SC) challenge with HPV16-Lucf PV in the peritoneal area [27, 28].

Three days later, all mice were anesthetized via intramuscular injection with 40 mg/kg of Zoletil50 (Virbac Laboratories, France), and 5 mg/kg of Rompun (Bayer Korea, Korea), followed by intravaginal pre-treatment with 20 μl of 4% Nonoxynol-9 (Sigma-Aldrich). The mice were injected intraperitoneally with luciferin (100 μl at 7 mg/ml)(Caliper Life Sciences, USA), and imaged for 10 min with an IVIS 200 bioluminescence imaging system (Xenogen, USA) to visualize the expression of luciferase by measuring the light emission. Equal areas encompassing the site of virus inoculation were analyzed using the Living Image 2.20 software (Xenogen, USA).

### Statistical Analysis

All statistical analyses were performed using GraphPad software version 7.0 (GraphPad Software). The significance of differences between treatment groups was analyzed via two-way analysis of variance (ANOVA) or two-tailed Student’s *t*-test. *P* values less than 0.05 were considered statistically significant.

## Results

### Screening of Anti-HPV Activities

**Fig. 1:**
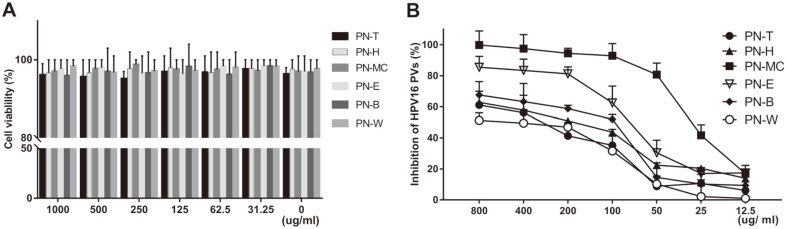
In vivo cytotoxicity and antiviral activity of pine needle extracts. (**A**) Cell viability was measured using the water-soluble tetrazolium salt (WST) method. 293TT cells were seeded on a 96-well plate at a density of 1 × 10^4^ cells/well, and cultured for 1 day, followed by treatment with serial dilutions of extracts and incubation at 37°C for 48 h. EZ-Cytox solution was added to each well and incubated for 2 h, followed by spectrophotometric measurement of absorbance at 540 nm. (**B**) The 293TT cells were pre-treated with each extract concentration (PN-T, PN-H, PN-MC, PN-E, PN-B, or PN-W) for 16 h. After washing with PBS, cells were infected with HPV16-SEAP PV (MOI of 20). SEAP activity was determined 48 h after infection and compared with untreated control. The experiment was performed in triplicate. Results are presented as the percentage of infection reduction and data are presented as means ± SD for three different samples (*p* < 0.001 for comparisons between each group).

In order to screen the antiviral activity of the pine needle extract against HPV, pine needles were extracted from each organic solvent. The cytotoxicity of pine needle extract in each organic solvent was measured in the 293TT cell line. None of the extracts showed cytotoxicity to 293TT cells at concentrations less than 1,000 μg/ml (Fig. 1A). The extracts were serially diluted from 800 μg/ml to 12.5 μg/ml and tested during pre-treatment. The results of HPV antiviral efficacy using PV showed that the activity of the organic solvent extract of pine needles was related to the concentration. PN-MC, PN-E, and PN-B inhibited more than 50% of HPV16 PV infections at 100 μg/ml of extract. In particular, PN-MC has been shown to inhibit HPV16 PV infection by more than 50% (EC_50_) at 25 μg/ml. Therefore, PN-MC was selected as an anti-HPV drug candidate for further study.

### Inhibitory Effect of PN-MC during Pre-Treatment

**Fig. 2:**
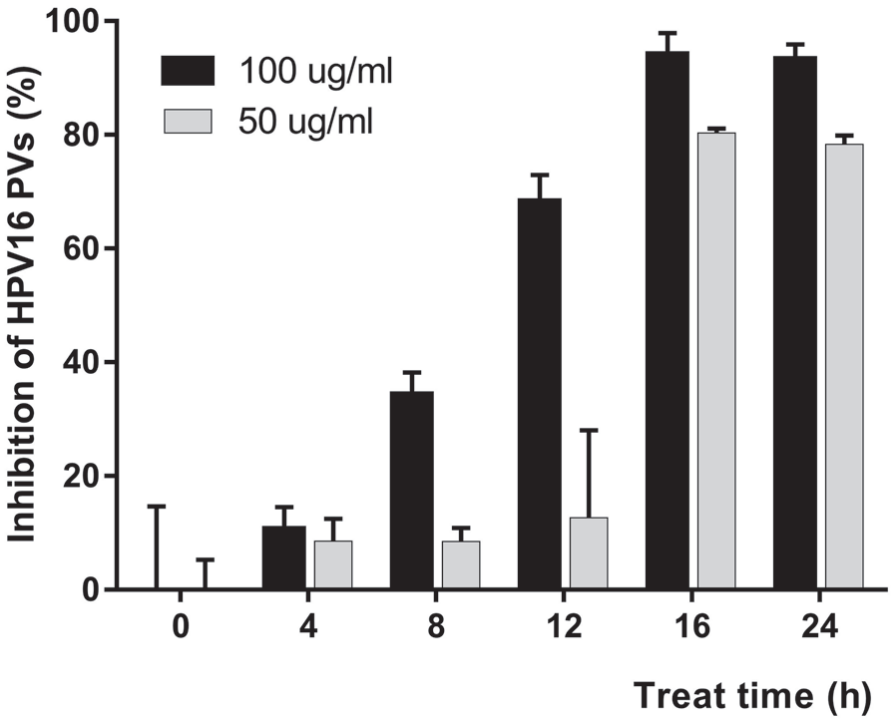
Inhibitory effects of pre-treatment. The 293TT cells were treated with PN-MC (100 μg/ml or 50 μg/ml) for 4, 8, 12, 16, or 24 h. After washing with PBS, cells were infected with HPV16-SEAP PVs (MOI of 20). SEAP activity was determined 48 h after infection and compared with untreated control. Results are expressed as the percentage of infection reduction and represent the mean of three independent experiments ± SD.

A pre-treatment assay was performed to assess the inhibitory activity of PN-MC extract on the entry of HPV into the 293TT cells. The cells were pre-treated with 100 μg/ml or 50 μg/ml of PN-MC for 4, 8, 12, 16, or 24 h to enable its interaction with the cell surface. The anti-HPV effect of 100 μg/ml of PN-MC treatment was observed in the group treated for 12 h or longer. Treatment with 50 μg/ml of PN-MC for 16 h and 24 h in the pre-treatment groups inhibited HPV16 PV by more than 80% (Fig. 2). Pre-treatment with HPV16 PV revealed that PN-MC effectively inhibited the viral entry by binding to the HPV receptor in the cells.

### Inhibition of Attachment between Virus and Cell

**Fig. 3:**
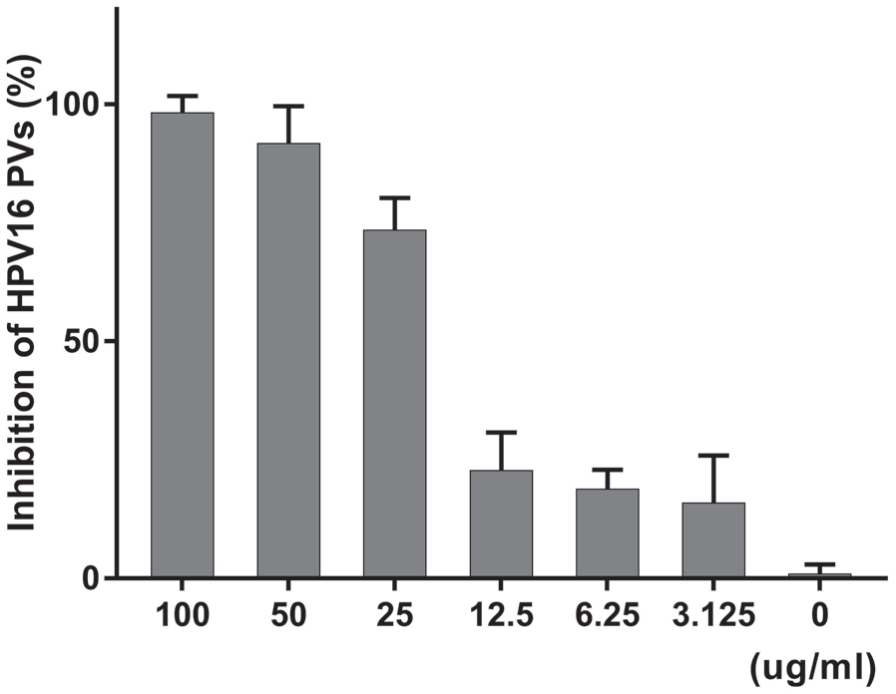
Inhibition of the attachment between virus and cell. Cells were pre-adsorbed with HPV16-SEAP PV at different concentrations of PN-MC extract (100 to 3.125 μg/ml) for 1 h at 4°C. Cells were washed twice with PBS and replaced with culture medium. After 48 h, the antiviral effect of the extract on the viral adsorption was evaluated using the SEAP Chemiluminescence Kit.

Various concentrations of PN-MC were mixed with HPV PV for 1 h to investigate the inhibitory efficacy of the virus against attachment to host cells. The mixture was then added to 293TT cells. The mixture containing PN-MC and HPV PV showed potent inhibition of HPV attachment at concentrations above 25 μg/ml (Fig. 3). The result shows that the pine needle extract PN-MC may also interact with HPV to block viral attachment to the cell.

### Post-Treatment Inhibitory Effect of PN-MC 

**Fig. 4:**
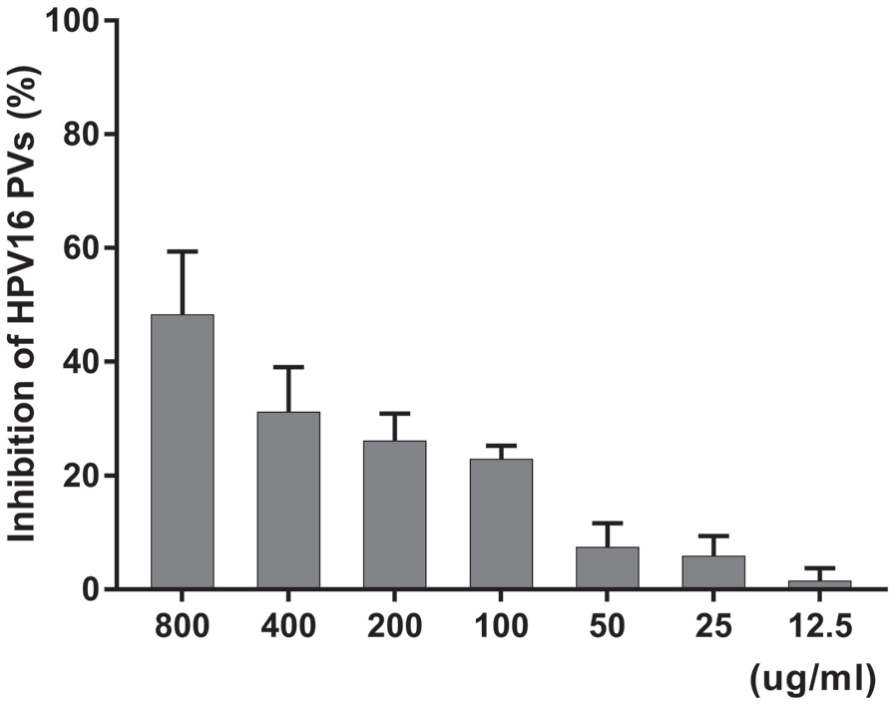
Antiviral effect of post-treatment. The cells were initially infected with HPV16-SEAP at a MOI of 20. After 1 h, the cells were washed with 200 μl of PBS, followed by the addition of different concentrations (800 to 12.5 μg/ml) of extract to the cells and then incubated for 48 h. After 48 h, the secreted alkaline phosphatase (SEAP) activity was measured with a luminescence counter.

Post-treatment assay was performed to evaluate whether PN-MC inhibited viral proliferation. Various concentrations of PN-MC were used for the treatment. In contrast to pre-treatment, the results of post-treatment showed no HPV inhibitory effect. Only a PN-MC concentration of 800 μg/ml showed 48.3% inhibition of HPV PVs (Fig. 4). As a result, it was concluded that PN-MC was not suitable as a therapeutic drug for HPV.

### In Vivo Antiviral Activity of PN-MC

**Fig. 5:**
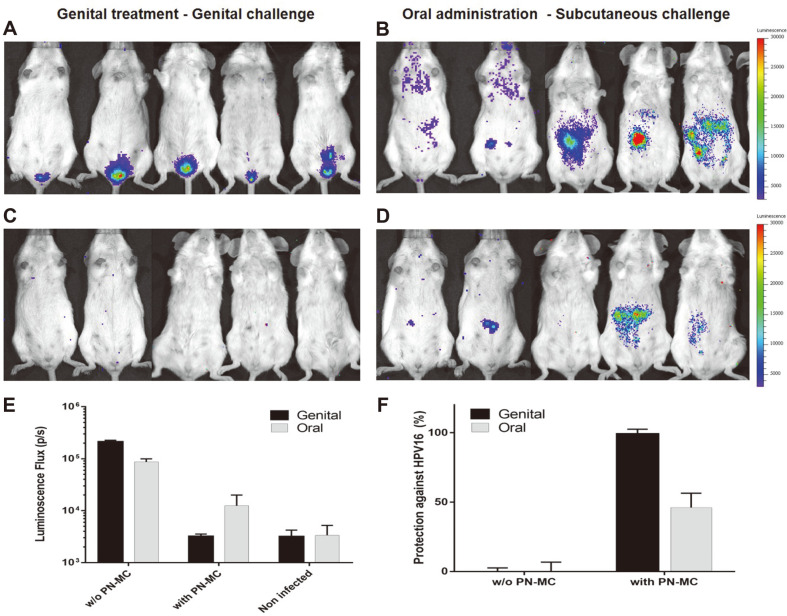
Protection against HPV16 PV challenge. The untreated mice received a genital (**A**) or subcutaneous (**B**) challenge with HPV16-Lucf PV. Genitally treated mice were challenged with HPV PV via genital tract (**C**) and orally administered mice were challenged subcutaneously (**D**). Mice were infected with 5 × 106 relative light units (RLU) of HPV16 PVs. Three days later, all mice were anesthetized and injected with luciferin into the genital tract or intraperitoneally. Molecular imaging analysis was performed for 10 min using an IVIS 200 bioluminescence imaging system (Xenogen, NJ, USA) to visualize the expression of luciferase by measuring light emission. Equal areas encompassing the sites of virus inoculation were analyzed (*p* < 0.001), and the bioluminescence was quantified in relative light units using Living Image 2.20 software (Xenogen, NJ, USA) (**E**). The mean values of protection rates were plotted using levels of inhibition in mice treated with PN-MC before challenge (**F**).

A murine model was used to evaluate the antiviral activity of PN-MC against HPV infection. Mice were pre-treated with PN-MC via genital or oral administration and then challenged with HPV PV to the genitals or subcutaneously, respectively. Mice treated via genitals showed complete protection against HPV. Fig. 5 shows that mice treated with PN-MC genitally did not show luciferase expression (perfect protection), whereas only 2 out of 5 were protected by oral treatment.

Figs. 5E and 5F showed the results of bioluminescence assay and the rate of protection against HPV PV. The negative control groups showed that the detection of PV via luminescence was significantly high (*p* < 0.001). Luminescence levels of genitally treated mice were 7-fold less than those of orally treated mice. At the levels of protection, genital treatment resulted in more than 97% protection against HPV PV. By contrast, in the case of SC infection after oral administration with PN-MC, the level of protection from HPV PV infection was 46%. Taken together, topical administration of PN-MC to the genitals inhibits HPV PV infection.

## Discussion

Here, we investigated the anti-HPV effect of pine needle extracts in vitro and in vivo. First, an in vitro antiviral assay was performed to determine the anti-HPV effects of various pine needle extracts. Based on the screening results, we selected PN-MC as a candidate agent with anti-HPV activity. Pre-treatment and cell adsorption of PN-MC effectively inhibited viral invasion. The results in the inhibition test of viral attachment to host cells indicate that PN-MC blocks viral attachment to cells by inhibiting the interaction with HPV and its receptors. However, post-treatment of extracts did not show a viral reduction. This phenomenon is related to the binding affinity of the extract to the HPV receptor, which appears to inhibit the entry rather than the viral replication. These results suggest that pine needle extract is an effective agent for preventing HPV infection, and therefore we used the PN-MC extract for pre-treatment in an animal model. During the in vivo test, mice were effectively protected against HPV when PN-MC was topically administered via the vaginal tract.

Previously, we isolated the compounds from the pine needle. Twelve compounds were isolated and registered in patent (composition for preventing and treating cervical cancer disease caused by HPV in several compounds obtained by fractionation from pine needle extract, US 2014/0363530). However, the yield of the compounds was so low that anti-HPV analysis could not be performed. Among the various organic extracts, large amounts of substances with anti-HPV function could be obtained in the methylene chloride layer.

Sexually transmitted diseases (STDs) can be transmitted via genital, orogenital, or anogenital contacts, and are a public health concern worldwide. Approximately one million people around the world are newly infected with STIs each day. Although several preventive strategies such as vaccination and a range of therapeutic drugs have drastically reduced the risk of contracting STIs, these infections continue to spread [29].

Although currently available HPV vaccines have significantly reduced the incidence of HPV infection, it is impossible to prevent the spread of more than 150 types of HPV. The HPV vaccine is an exceptional primary prevention tool, but the question of adequate secondary prevention strategies remains open. Topical virucides have been proposed and pursued as a secondary prevention strategy [30]. Other studies have suggested candidates for topical microbicides formulated as gels, creams, and films to inhibit STI transmission, including Nonoxynol-9, Tenofovir, and Carrageenan [31].

A number of important factors such as safety, efficacy, cost, acceptability, efficiency, drug delivery patterns, and tolerance primarily influence the development of virucides. A variety of plant extracts showed proven antiviral activity. Optimizing plant-based antiviral agents requires a better understanding of the structure and antiviral mechanism of various extracts. Our results also provide insight into viral treatment using plant extract. It can be concluded that pine needle extracts exhibit antiviral effect against HPV. In addition, due to the abundance of pine needles available as a natural resource, the production cost is relatively low compared to other chemically synthesized drugs. The low cost of microbicides from PN-MC provides a better approach to popular prevention.

In conclusion, treatment with the PN-MC extract derived from the pine needles reduced HPV in vitro and in a mouse model. Therefore, the findings indicate that the PN-MC extract can be used as a preventive agent against HPV and developed as an active ingredient in topical virucides against HPV infections. Further studies are needed to determine the precise antiviral mechanism of PN-MC, develop appropriate PN-MC formulations, and administer topical treatments to inhibit HPV.

## References

[1] Bray F, Ferlay J, Soerjomataram I, Siegel RL, Torre LA, Jemal A (2018). Global cancer statistics 2018: GLOBOCAN estimates of incidence and mortality worldwide for 36 cancers in 185 countries. CA Cancer J. Clin..

[2] Lembo D, Donalisio M, Rusnati M, Bugatti A, Cornaglia M, Cappello P (2008). Sulfated K5 Escherichia coli polysaccharide derivatives as wide-range inhibitors of genital types of human papillomavirus. Antimicrob. Agents Chemother..

[3] Small W, Bacon MA, Bajaj A, Chuang LT, Fisher BJ, Harkenrider MM (2017). Cervical cancer: A global health crisis. Cancer.

[4] Li N, Franceschi S, Howell-Jones R, Snijders PJ, Clifford GM (2011). Human papillomavirus type distribution in 30,848 invasive cervical cancers worldwide: Variation by geographical region, histological type and year of publication. Int. J. Cancer.

[5] Donalisio M, Rusnati M, Civra A, Bugatti A, Allemand D, Pirri G (2010). Identification of a dendrimeric heparan sulfate-binding peptide that inhibits infectivity of genital types of human papillomaviruses. Antimicrob. Agents Chemother..

[6] Stanley MA (2012). Genital human papillomavirus infections: current and prospective therapies. J. Gen. Virol..

[7] Moscicki AB (2005). Impact of HPV infection in adolescent populations. J. Adolesc. Health.

[8] Wang SX, Zhang XS, Guan HS, Wang W (2014). Potential anti-HPV and related cancer agents from marine resources: an overview. Mar. Drugs.

[9] Cha MK, Lee DK, An HM, Lee SW, Shin SH, Kwon JH (2012). Antiviral activity of *Bifidobacterium* adolescentis SPM1005-A on human papillomavirus type 16. BMC Med..

[10] Underwood MR, Shewchuk LM, Hassell AM, Phelps WC (2000). Searching for antiviral drugs for human papillomaviruses. Antivir. Ther..

[11] Ahn WS, Yoo J, Huh SW, Kim CK, Lee JM, Namkoong SE (2003). Protective effects of green tea extracts (polyphenon E and EGCG) on human cervical lesions. Eur. J. Cancer Prev..

[12] Fradet-Turcotte A, Archambault J (2007). Recent advances in the search for antiviral agents against human papillomaviruses. Antivir. Ther..

[13] Lee BH, Chathuranga K, Uddin MB, Weeratunga P, Kim MS, Cho WK (2017). Coptidis Rhizoma extract inhibits replication of respiratory syncytial virus in vitro and in vivo by inducing antiviral state. J. Microbiol..

[14] Iljazovic E, Ljuca D, Sahimpasic A, Avdic S (2006). Efficacy in treatment of cervical HRHPV infection by combination of beta interferon, and herbal therapy in woman with different cervical lesions. Bosn. J. Basic Med. Sci..

[15] Polansky H, Itzkovitz E, Javaherian A (2017). Human papillomavirus (HPV): systemic treatment with Gene-Eden-VIR/Novirin safely and effectively clears virus. Drug Des. Devel. Ther..

[16] Miyoshi N, Tanabe H, Suzuki T, Saeki K, Hara Y (2020). Applications of a standardized green tea catechin preparation for viral warts and human papilloma virus-related and unrelated cancers. Molecules.

[17] Ha TKQ, Lee BW, Nguyen NH, Cho HM, Venkatesan T, Doan TP (2020). Antiviral activities of compounds isolated from pinus densiflora (Pine Tree) against the influenza A Virus. Biomolecule.

[18] Zou K, Zhao Y, Tu G, Cui J, Jia Z, Zhang R (2000). Two diastereomeric saponins with cytotoxic activity from *Albizia julibrissin*. Carbohydr. Res..

[19] Buck CB, Pastrana DV, Lowy DR, Schiller JT (2004). Efficient intracellular assembly of papillomaviral vectors. J. Virol..

[20] Pastrana DV, Buck CB, Pang YY, Thompson CD, Castle PE, FitzGerald PC (2004). Reactivity of human sera in a sensitive, highthroughput pseudovirus-based papillomavirus neutralization assay for HPV16 and HPV18. Virology.

[21] Buck CB, Cheng N, Thompson CD, Lowy DR, Steven AC, Schiller JT (2008). Arrangement of L2 within the papillomavirus capsid. J. Virol..

[22] Buck CB, Thompson CD (2007). Production of papillomavirus-based gene transfer vectors. Curr. Protoc. Cell Biol..

[23] Johnson KM, Kines RC, Roberts JN, Lowy DR, Schiller JT, Day PM (2009). Role of heparan sulfate in attachment to and infection of the murine female genital tract by human papillomavirus. J. Virol..

[24] Buck CB, Thompson CD, Pang YY, Lowy DR, Schiller JT (2005). Maturation of papillomavirus capsids. J. Virol..

[25] Conway MJ, Alam S, Ryndock EJ, Cruz L, Christensen ND, Roden RB (2009). Tissue-spanning redox gradient-dependent assembly of native human papillomavirus type 16 virions. J. Virol..

[26] Lee HJ, Hur YK, Cho YD, Kim MG, Lee HT, Oh YK (2012). Immunogenicity of bivalent human papillomavirus DNA vaccine using human endogenous retrovirus envelope-coated baculoviral vectors in mice and pigs. PLoS One.

[27] Roberts JN, Buck CB, Thompson CD, Kines R, Bernardo M, Choyke PL (2007). Genital transmission of HPV in a mouse model is potentiated by nonoxynol-9 and inhibited by carrageenan. Nat. Med..

[28] Longet S, Schiller JT, Bobst M, Jichlinski P, Nardelli-Haefliger D (2011). A murine genital-challenge model is a sensitive measure of protective antibodies against human papillomavirus infection. J. Virol..

[29] Singh O, Garg T, Rath G, Goyal AK (2014). Microbicides for the treatment of sexually transmitted HIV Infections. J. Pharm. (Cairo)..

[30] Calagna G, Maranto M, Paola C, Capra G, Perino A, Chiantera V (2020). Secondary prevention' against female HPV infection: literature review of the role of carrageenan. Expert Rev. Anti. Infect. Ther..

[31] Lee C (2020). Carrageenans as broad-spectrum microbicides: currentstatus and challenges. Mar. Drugs.

